# Developmental mechanisms of CPSP: Clinical observations and translational laboratory evaluations

**DOI:** 10.1080/24740527.2021.1999796

**Published:** 2021-12-29

**Authors:** Suellen M. Walker

**Affiliations:** Clinical Neurosciences (Pain Research), Developmental Neurosciences, UCL GOS Institute of Child Health, London, UK; Department of Paediatric Anaesthesia and Pain Medicine, Great Ormond Street Hospital NHS Foundation Trust, London, UK

**Keywords:** pain, surgery, children, nociception, developmental neurobiology, somatosensory phenotype

## Abstract

Understanding mechanisms that underly the transition from acute to chronic pain and identifying potential targets for preventing or minimizing this progression have specific relevance for chronic postsurgical pain (CPSP). Though it is clear that multiple psychosocial, family, and environmental factors may influence CPSP, this review will focus on parallels between clinical observations and translational laboratory studies investigating the acute and long-term effects of surgical injury on nociceptive pathways. This includes data related to alterations in sensitivity at different points along nociceptive pathways from the periphery to the brain; age- and sex-dependent mechanisms underlying the transition from acute to persistent pain; potential targets for preventive interventions; and the impact of prior surgical injury. Ongoing preclinical studies evaluating age- and sex-dependent mechanisms will also inform comparative efficacy and preclinical safety assessments of potential preventive pharmacological interventions aimed at reducing the risk of CPSP. In future clinical studies, more detailed and longitudinal peri-operative phenotyping with patient- and parent-reported chronic pain core outcomes, alongside more specialized evaluations of somatosensory function, modulation, and circuitry, may enhance understanding of individual variability in postsurgical pain trajectories and improve recognition and management of CPSP.

## RÉSUMÉ

La compréhension des mécanismes qui sous-tendent la transition de la douleur aiguë à la douleur chronique et la détermination de cibles potentielles pour prévenir ou minimiser cette progression ont une pertinence particulière pour la douleur chronique. La détermination de cibles potentielles pour prévenir ou minimiser cette progression sont particulièrement pertinentes pour la douleur postopératoire chronique (DPOC). Bien qu'il soit clair que de multiples facteurs psychosociaux, familiaux et environnementaux peuvent influencer la DPOC, cette revue se concentrera sur les parallèles entre les observations cliniques et les études translationnelles en laboratoire qui étudient les effets aigus et à long terme d'une blessure chirurgicale sur les voies nociceptives. Cela inclut les données relatives aux altérations de la sensibilité à différents points le long des nociceptives, de la périphérie au cerveau; les mécanismes dépendant de l'âge et du sexe qui sous-tendent la transition de la douleur aiguë à la douleur persistante; les cibles potentielles des interventions préventives et l'impact d'une blessure chirurgicale antérieure. Les études précliniques en cours, qui évaluent les mécanismes dépendant de l'âge et du sexe, permettront également d'évaluer l'efficacité comparative et la sécurité préclinique d'éventuelles interventions pharmacologiques préventives potentielles visant à réduire le risque de DPOC. Dans les futures études cliniques, un phénotypage péri-opératoire plus détaillé et longitudinal avec des résultats rapportés par les patients - et les parents- ainsi que des évaluations plus spécialisées de la fonction de la modulation et des circuits somatosensoriels, pourrait améliorer la compréhension de la variabilité individuelle des trajectoires de la douleur postopératoire et améliorer la reconnaissance et la gestion de la DPOC.

## Introduction

Understanding mechanisms that underly the transition from acute to chronic pain and identifying potential targets for preventing or minimizing this progression are high priorities for pain research^1,2^ and have specific relevance for persistent postsurgical pain.^3^ Though enhanced sensitivity of nociceptive mechanisms provides a warning of acute injury and is adaptive, pain that persists beyond the expected period of recovery can be associated with significant pain-related disability and mediated at multiple points along nociceptive pathways. Chronic pain may reflect ongoing excitation, decreased inhibition or inability to recruit endogenous inhibition, failure of active resolution mechanisms (e.g., resolvins and interleukin 10), or unmasking of hyperalgesia related to prior exposures or injuries.^2,4–6^

Nociceptive mechanisms and responses to injury and analgesia vary throughout postnatal development.^7^ Laboratory studies can evaluate mechanisms underlying clinical observations in children with chronic postsurgical pain (CPSP) by (1) quantifying alterations in sensitivity at different points along nociceptive pathways from the periphery to brain, (2) evaluating age- and sex-dependent mechanisms underlying the transition from acute to persistent pain, (3) identifying potential therapeutic targets and comparing the safety and efficacy of interventions instituted before surgery (i.e., preventive) and/or after injury (i.e., during maintenance phase when hypersensitivity is established), and (4) determining the impact of prior exposures, such as different forms of stress and injury. This review will focus on parallels between clinical observations and related evaluations in translational laboratory studies to investigate the mechanisms and pathophysiology of CPSP.

## Laboratory Models of Surgical Injury

Because pain following surgery may be related to skin incision, muscle injury, inflammation, and/or nerve injury,^8^ specific models have been developed to investigate acute and persistent alterations in nociceptive processing following surgical injury.^9^ Specific contributions of additional factors that can contribute to, or exacerbate, pain following surgery (e.g., peripheral inflammation, full-thickness skin wound, visceral injury, immune challenge, traumatic nerve injury, stress) can also be evaluated at different postnatal ages. Studies performed in rodents allow comparison of age- and sex-dependent injury effects across different stages of mammalian development.^10^ Many evaluations have focused on initial injury in the neonatal period and early infancy (first one to two weeks of postnatal life in rodents) because responses in the developing nervous system may differ from those at older ages, but associated persistent changes in somatosensory function can have an impact on the response to subsequent injury into adulthood.^11^

Plantar hind paw incision incorporates incision of the skin and underlying plantaris muscle and produces cellular and molecular alterations specific to this type of injury.^6,12,13^ Acute behavioral hyperalgesia (reduced hindlimb reflex thresholds) is evoked, with the degree and duration of sensitivity influenced by postnatal age^14,15^ but not sex.^16^ In younger animals, incision-induced electrophysiological changes in large dorsal root ganglion neurons persist beyond the period of behavioral hyperalgesia, afferent-evoked activity in second-order dorsal horn neurons is more marked and rapid,^17,18^ and noxious-evoked potentials in the somatosensory cortex are enhanced and more resistant to increasing isoflurane anesthesia.^19^ In addition, incision produces developmentally regulated long-term changes in nociceptive processing and response to re-incision^20,21^ that differ from other injury models (see reviews for inflammatory, nerve injury, arthritis, stress, and immune challenge models^7,11,22–28^).

Laparotomy in newborn mice^29^ and skin/muscle incision on the thigh (modified from the adult skin/muscle incision and retraction model^30^) at postnatal day (P)3 are associated with persistent alterations in sensory thresholds and in the latter with an enhanced degree and duration of hyperalgesia following re-incision of the ipsilateral hind paw.^16^ Rodent models of inguinal hernia repair^31,32^ and thoracotomy^33^ have not been assessed in juvenile animals.

## Developmental Mechanisms of Acute and Chronic Surgical Pain

Pain and hyperalgesia following surgical injury involve alterations in peripheral and central nociceptive pathways.^6,9^ In clinical studies, this can be evaluated with a range of techniques during or after surgery that include, but are not limited to, measurement of reflex thresholds,^34^ somatosensory testing,^35–37^ physiological reactivity and evoked responses (e.g., stress hormones,^38^ inflammatory markers, monitors of autonomic tone^39^), and brain responses with electroencephalography and neuroimaging.^40–42^ Though these measures may identify children and adolescents with enhanced sensitivity in the perioperative period or delayed recovery, further longitudinal studies are required to assess feasibility and utility at different ages and evaluate associations with the degree and/or risk of CPSP.

Nociceptive mechanisms contributing to persistent pain can include cellular plasticity with changes in molecular profile and translation regulation that shift the nociceptor toward hyperexcitability, alterations in neuronal circuitry and activity, systems-level changes such as immune cell recruitment or cell proliferation, and organism-level effects related to comorbidities and affective, behavioral, and motivational changes.^2^

### Peripheral and Spinal Mechanisms

Acute peripheral hyperalgesia following surgery has been quantified by changes in sensory withdrawal thresholds in human infants.^34^ At older ages, quantitative sensory testing (QST) has identified altered sensitivity and dynamic allodynia adjacent to neonatal surgical scars in extremely preterm-born children and young adults^43,44^ and punctate hyperalgesia many years following childhood surgery.^45–47^ Associations between persistent alterations in scar-related sensitivity and the degree or duration of pain if subsequent surgery is required in the same region require further evaluation.

Peripheral nociceptors respond to noxious stimuli following birth, and primary hyperalgesia in the region of plantar hind paw incision has been demonstrated across a range of postnatal ages in rodents.^14,15^ Surgical injury will also evoke a peripheral inflammatory response that can contribute to acute hyperalgesia at all postnatal ages, with longer-term alterations in sensitivity varying with the type and degree of insult.^48,49^

Growth hormone signaling influences activity and development of peripheral nociceptive neurons in neonatal rodents, and a detailed series of experiments following dorsal hind paw and muscle incision identified sequestration of growth hormone by infiltrating macrophages and upregulation of transcription factors related to excitatory receptors and channels that lead to nociceptor sensitization.^50^ Hyperinnervation following full-thickness skin wounding may contribute to persistent sensitivity and is more pronounced at younger ages due to differences in trophic and nerve guidance factors (nerve growth factor,^51^ neurotrophin 3,^52^ ephrin signaling^53^) and is also seen following plantar incision.^11^

Afferent input induces central sensitization in the spinal cord, with increased excitation and/or impaired inhibition.^6,54^ Mechanisms can include changes in synaptic function, reduced local and/or descending inhibitory effects, and potential maladaptive and long-term changes due to translational effects on gene expression.^54^ Developmental changes in spinal cord structure and function, including larger and overlapping cutaneous receptive fields and a relative excess of excitatory and delayed maturation of inhibitory synaptic signaling, contribute to low reflex thresholds.^20,55^ Hind paw incision in neonatal rodents induces a range of age-dependent acute and persistent alterations in spinal cord synaptic function, including increased excitatory signaling and reduced inhibitory transmission, alterations in receptor expression, ion channel function, and differential gene expression^56^ (see Brewer and Baccei’s^20^ review for details).

Clinically, preventive interventions for postsurgical pain focus on reducing afferent input (e.g., local anesthetic blockade), reducing excitation (e.g., N-methyl-d-aspartate antagonist), or enhancing inhibition (e.g., gabapentinoids).^57–59^ In rodents, neonatal peri-incision sciatic blockade has preventive analgesic effects (lack of hyperalgesia at 24 h),^15,60^ whereas opioids block hyperalgesia only during the duration of action of the drug and sensitivity at later time points does not differ from saline controls.^61^ More specific targeting of mechanisms that underlie the transition from acute to persistent pain or that enhance endogenous pain resolution mechanisms may more specifically reduce the risk of CPSP,^1^ but efficacy and safety require evaluation in preclinical models at different stages of postnatal development.

### Descending Modulation

**Figure 1.**
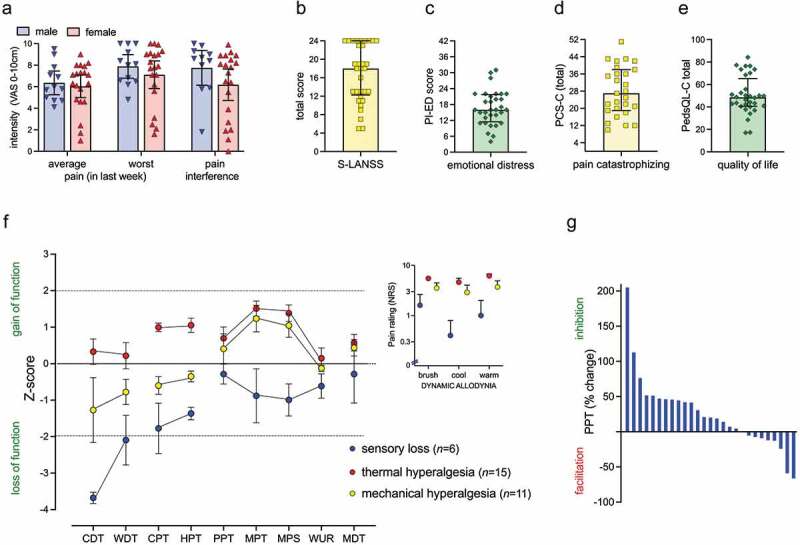
Characteristics and impact of neuropathic CPSP. (a) Neuropathic CPSP is graded as moderate–severe intensity in both male (*n* = 12) and female (*n* = 20) adolescents (median [interquartile range] age: 15 [12.9, 16.5] years) and interferes with normal activity. (b) Total scores on the Self-report Leeds Assessment of Neuropathic Symptoms and Signs screening tool in the majority of adolescents above the cutoff for identification of neuropathic pain in adults (score of 12 or above in 25/32, 78%). (c) Increased anxiety and depression is reflected by Pediatric Index of Emotional Distress scores (16–20, mild; 22–28, moderate). (d) Pain Catastrophizing Scale for Children scores are increased (15–25, moderate; 26 and above, severe). (e) Impaired quality of life in school, physical, emotional, and social domains (Pediatric Quality of Life Inventory–Child Scale) is reflected by low total scores (<78, mild; <70 severe). (g) Quantitative sensory testing with a range of modalities identified distinct sensory profiles. Individual patient pain site thresholds were converted into *z*-scores calculated with reference to within-cohort body region–specific control data. The *z*-score plot for each individual patient was grouped according to the closest matching mechanism-related sensory profiles identified in adults: sensory loss (*n* = 6), thermal hyperalgesia (*n* = 15), or mechanical hyperalgesia (*n* = 11). Dynamic allodynia to brush, cool (25°C) and warm (40°C) rollers in the region of pain was rated on a 0 to 10 numerical rating scale. (h) Conditioned pain modulation was assessed with a cold conditioning stimulus (immersion of hand in 5°C water bath) and variable test stimulus (change in contralateral knee pressure pain threshold). CPM effect (% change from baseline pressure pain threshold at 15 s) shows a spectrum of individual responses, with a shift to facilitation in 8/27 adolescents. CDT, cold detection threshold; WDT, warm detection threshold; CPT, cold pain threshold; HPT, heat pain threshold; PPT, pressure pain threshold; MPT, mechanical pain threshold; MPS, mechanical pain sensitivity; WUR, wind-up ratio; MDT, mechanical detection threshold. Data for CPSP subgroup extracted from Verriotis et al.^35^; see full manuscript^28^for further details of project registration (clinicaltrials.gov NCT03312881), methodology, parental consent and participant consent/assent was obtained and ethics approval was gained from National Health Service West Midlands-Black Country Research Ethics Committee (Ref: 17/WM/0306; Approval Date: 23-8-2017).

The balance between descending inhibition and facilitation can be assessed in clinical populations with conditioned pain modulation (CPM).^62^ In adults, inhibition is the usual baseline response, and reduced inhibitory CPM before surgery predicted CPSP.^59,63^ In adolescents with idiopathic scoliosis, reduced inhibitory modulation or a shift to facilitation was seen in 21% and 28% respectively,^64^ but potential links with risk of CPSP require evaluation in longitudinal perioperative studies. In adolescents with established neuropathic CPSP, robust inhibition was identified in only 44%, and 30% demonstrated a facilitatory response (Figure 1).^35^ Though identifying impaired CPM preoperatively may inform risk and individualized therapy,^36,63^ age-dependent effects also need to be considered because the degree of inhibitory CPM is reduced at younger ages (8–11 vs. 12–17 years.).^65^

Descending modulation is mediated by brainstem centers (e.g., periaqueductal gray, rostroventral medulla, locus coeruleus) that receive input from higher centers (e.g., amygdala and limbic system) and have descending projections that can inhibit or facilitate spinal excitability.^66^ In adult rodents, nociceptive C-fiber input is tonically inhibited (although this shifts to facilitation following injury), whereas at younger ages there is a relative excess of facilitation.^67,68^ The delayed maturation of inhibitory mechanisms is influenced by endogenous opioid and endocannabinoid signaling,^69^ and surgical injury in neonatal rodents produces long-term changes in the balance of inhibitory/facilitatory modulation.^70^

### Brain Structure and Circuits

Clinical neuroimaging studies have evaluated changes in structure, connectivity, or blood flow in brain regions associated with attention, sensory/discriminative, and affective/motivational aspects of pain experience^71–74^ and also identified predictors of the transition from acute to persistent pain.^75^ In adolescents with complex regional pain syndrome, alterations in brain structure and connectivity have been linked with self-reported outcomes (e.g., amygdala circuits and fear of pain) that improved following interdisciplinary treatment.^76,77^ Magnetic resonance imaging is feasible in children with moderate–severe persistent postsurgical pain,^78^ but pre- and postsurgery studies are yet to identify risk factors for CPSP in children, and effects on brain structure associated with prior neonatal surgery and intensive care also require consideration.^43,79,80^

Nociceptive circuits in the brain and central responses to noxious and innocuous stimuli undergo significant changes in the postnatal period, and activity-dependent maturation of circuits may be altered by injury.^20^ In infant rodents, evoked electroencephalograph responses in the somatosensory cortex were rapidly sensitized by hindpaw incision.^19^ Following hind paw surgical injury in adult rodents, functional magnetic resonance imaging identified evoked responses in brain regions involved in sensory/discriminative, affective/attentional, and descending modulation,^81^ and incision-induced reductions in the volume of different brain regions (anterior cingulate cortex, amygdala, thalamus, corpus callosum) were also influenced by early life stress and maternal separation.^82^

## Nerve Injury and Neuropathic CPSP

CPSP is often associated with characteristics of neuropathic pain in adults.^83,84^ Although multiple mechanisms including inflammation and pain arising from muscles, joints, and viscera may also contribute to CPSP,^85^ this section will focus on neuropathic pain, because this can be difficult to recognize in children and has specific implications for management.^86^ In pediatric studies, there is significant variability in the diagnostic criteria and reported prevalence of neuropathic CPSP (e.g., 10%–89% with CPSP following scoliosis surgery^87–89^). Outcomes used to support possible or probable neuropathic pain have included history and clinical descriptors, neuropathic screening tool questionnaires, somatosensory testing, conditioned pain modulation, and response to treatment (e.g., topical lidocaine patch)^35,87,88–91^. In our tertiary pediatric pain clinic cohort of adolescents with peripheral neuropathic pain (based on clinical features, sensory descriptors, screening tool, and somatosensory testing), CPSP accounted for 32/52 (62%) cases and was associated with moderate–severe pain intensity (Figure 1a) and significant pain-related disability (Figures 1b–1e).^35^ QST identified dynamic allodynia and distinct sensory profiles in the region of pain and prior surgical scars (Figure 1f) that have parallels with QST findings in adults.^92^ Rather than being specific to the etiology of neuropathic pain, underlying mechanisms and relative efficacy of neuropathic medications may be more closely related to the different sensory profiles: sensory loss (denervation and spontaneous pain due to ectopic action potentials proximal to injured nociceptors; response to antidepressants), thermal hyperalgesia (peripheral sensitization with low threshold and spontaneous activity in “irritable nociceptors”; predicted efficacy with a sodium channel blocker, such as lidocaine patch and moderate response to antidepressant or gabapentinoid), and mechanical hyperalgesia (sensitization and spontaneous activity in peripheral and/or central nervous system and predicted efficacy with gabapentinoids).^92,93^

In rodents, responses to traumatic nerve injury vary throughout postnatal development, with a delayed onset of allodynia following nerve injury at younger ages that has been associated with a switch from an anti-inflammatory to pro-inflammatory response in the spinal cord.^23^ Delayed emergence of neuropathic pain has also been reported following traumatic or surgical nerve injury in children.^35,86,94^ The potential for preclinical studies to also link specific sensory modalities and mechanisms to efficacy of pharmacological interventions has been highlighted^95^ and warrants further assessment at different developmental stages. In addition, more complex behavioral tasks in adult rodents have evaluated alterations in motivational–affective response (e.g., conditioned place preference, social interaction, anxiety) and cognitive function (e.g., memory and attention)^95–98^ and have identified long-term effects following surgical injury in early life (increased anxiety, impaired attentional performance and learning^99^).

## Impact of Prior Injury

Prior surgery is a risk factor for CPSP in adults,^59^ and repeated surgery is not uncommon during childhood. Surgery may be required in early life for congenital anomalies and complications of prematurity, and 20% of 5609 neonates recruited from 31 European countries required repeat surgery prior to 60 weeks postmenstrual age.^100^ In infants, repeat surgery in the same dermatome as prior neonatal surgery was associated with increased perioperative pain and stress.^38^ A study recruiting 8- to 18-year-olds for evaluation of CPSP noted that 148/237 (61%) had undergone previous surgery and 148 (62%) had an ongoing pain problem prior to surgery.^101^ In our cohort of 32 adolescents with neuropathic CPSP, 40% had required multiple surgeries throughout childhood, and pain had only become persistent after the most recent surgery in 20%.^35^ Multiple psychosocial, environmental, and genetic factors can influence pain experience and the transition from acute to chronic pain,^1,2,102^ and prior hospitalization may influence psychosocial factors such as anxiety and catastrophizing that are associated with increased risk of CPSP.^101,103^ Because the response to surgery and nerve injury varies with age and surgery during childhood has been associated with persistent alterations in sensory processing, a past history of prior surgery and pain exposures is relevant for the clinical assessment of CPSP and evaluation of underlying mechanisms. Despite undergoing the same surgery, only a proportion of patients develop CPSP, and not all children and adults with persistent sensory changes related to surgical scars have associated pain. Therefore, in addition to identifying factors that increase the risk of CPSP, factors associated with improved recovery and resilience need to be evaluated.^104,105^

Epidural, regional, and systemic local anesthetic administration reduces the risk of chronic postsurgical pain in adults,^59^ particularly following thoracotomy, breast cancer surgery, and cesarean section.^106^ One pediatric study met the inclusion criteria (single study following pectus excavatum surgery^107^ not included in meta-analysis), and the authors^106^ highlighted the need for larger, high-quality studies assessing the impact of regional anesthesia on CPSP in children. An increasing range of local anesthetic techniques are utilized to reduce perioperative pain in children,^59,108^ and laboratory studies demonstrate both acute perioperative and long-term benefit on the response to repeat surgery. Use of peripheral, regional, and potential long-acting preparations and catheter techniques may have additional benefit in children to both reduce the risk of CPSP and also minimize the impact of early life surgery on subsequent injury response.

Prior surgery may have triggered biological changes that contribute to CPSP, because nociceptive pathways can be “primed” by noxious input/injury. Early life injury has been associated with a range of developmentally regulated peripheral and spinal cord mechanisms that contribute to an enhanced degree and duration of hyperalgesia following subsequent re-incision in adulthood.^11,20^ In rodents, reducing primary afferent input by sciatic nerve blockade at the time of neonatal hind paw incision prevents persistent changes in synaptic signaling.^109^ In addition, pre- and postincision neonatal sciatic block prevents the enhanced response to re-incision in adulthood (incision-induced hyperalgesia does not differ from animals without a prior injury),^61^ whereas neonatal opioids do not have this long-term effect (enhanced re-incision hyperalgesia does not differ from control animals receiving saline at the time of neonatal incision).^61^ As noted above, peripheral growth hormone signaling is altered by neonatal incision, and injection of growth hormone into hind paw muscles at the time of neonatal incision prevented the enhanced re-incision response in adulthood.^50^

Hyperalgesic priming is a form of plasticity in primary afferent nociceptive fibers.^110^ Prior exposure to an inflammatory stimulus (e.g., hind paw injection of carrageenan or interleukin 6) or hind paw incision results in more prolonged hyperalgesia following a subsequent challenge several weeks later with a different or previously subthreshold mediator.^111^ Targeting this form of plasticity may reduce the transition to persistent pain,^111,112^ but sex-dependent effects need to be considered because mechanisms and response to preventive interventions differ in adult male and female rodents.^113–115^

Tissue injuries, including surgical incision, activate endogenous inhibitory mechanisms in the spinal cord and brain that contribute to the resolution of pain but may also mask ongoing hypersensitivity (i.e., latent sensitization).^116,117^ Pathways include endogenous opioid signaling, with subsequent opioid antagonist administration unmasking the hypersensitivity, and long-term alterations in the constitutive activity of the mu opioid receptor increasing excitatory actions via altered N-methyl-d-aspartate receptor function.^118^ Activity at mu opioid receptors and kappa opioid receptors, but not delta opioid receptors, modulates latent sensitization following hind paw incision, and kappa opioid receptor–mediated inhibition of latent sensitization was greater in females.^119^ Similarly, in human adult volunteers, high-dose naloxone unmasks hyperalgesia following an experimental thermal injury.^120,121^ Mechanism-based preventive interventions are effective in adults,^2^ but because the acute and long-term effects of opioid exposure vary with postnatal age and sex,^122,123^ and when opioid is administered in the presence or absence of surgical injury,^61^ evaluation at younger ages is also required.^122,123^

Neuroimmune interactions involving neurons, microglia, astrocytes, and T cells contribute to injury-induced changes in sensitivity and persistent pain.^124,125^ However, neuroimmune signaling is sexually dimorphic.^56,126,127^ Microglial inhibitors in male, but not female, adult rodents reduce pain behaviors following peripheral nerve injury,^128,129^ hind paw inflammation,^130^ and hyperalgesic priming,^131^ whereas T cells may play key modulatory roles in females.^132^ Microglia can be primed by early life exposures, resulting in intrinsic phenotypic changes and an exaggerated response to subsequent challenges.^133,134^ Neonatal surgical incision primes spinal microglia, resulting in an enhanced degree and duration of microglial reactivity following re-incision in later life, and the associated enhanced hyperalgesia is reduced by microglial inhibitors in adult males.^135,136^ Preventive, but sex-dependent, effects are also evident from early development, because microglial inhibitors at the time of neonatal incision prevented the enhanced response to adult re-incision in males but not females.^16^ Microglia also have important roles in developing neural circuits (e.g., neurogenesis, synaptic pruning, and synaptic plasticity),^137,138^ and perinatal insults can alter the normal sex-dependent trajectory of microglial development.^139^ Therefore, alongside the well-documented persistent alterations in excitatory and inhibitory signaling induced by neonatal incision,^20^ microglia may also influence developing spinal circuits.

## Summary

The prevalence and impact of CPSP in children and adolescents is increasingly recognized. Detailed peri-operative phenotyping and longitudinal follow-up will improve prediction of CPSP and identify targets for intervention. Alongside patient- and parent-reported measures that encompass recommended domains of core outcomes for pediatric chronic pain,^140^ specialized evaluations of somatosensory function and pain modulation, and assessments of connectivity and activity in central pain circuits, will contribute to understanding variability in postoperative pain trajectories. Ongoing translational preclinical studies evaluating age- and sex-dependent mechanisms will also inform comparative efficacy and preclinical safety assessments of potential preventive pharmacological interventions.
